# Herpes Simplex Virus Type 2 Encephalitis in an Immunocompetent Adult: A Case Report on an Unusual but Relevant Cause of Significant Neurological Morbidity

**DOI:** 10.7759/cureus.25968

**Published:** 2022-06-15

**Authors:** Ashish Jain, Khandakar M Hussain, Yazeed G Sweedan, Muhammad Ali Raza, Maha Mumtaz

**Affiliations:** 1 Internal Medicine, Conemaugh Memorial Medical Center, Johnstown, USA

**Keywords:** viral encephalitis, viral meningitis, hsv meningitis, hsv-2, acute encephalitis

## Abstract

Encephalitis refers to inflammation of the brain that is most frequently caused by viral infection (particularly herpes simplex virus type 1 [HSV-1]). In some instances, it may be associated with substantial neurological mortality and long-term morbidity. Although HSV-1 is the most common agent involved in producing neurological infections and disorders, herpes simplex virus type 2 (HSV-2) can occasionally affect the central nervous system, particularly in immunocompromised patients. We discuss the case of an immunocompetent male patient with a history of well-controlled diabetes who presented with symptoms of encephalitis. Our patient did not have a history of herpes infection, indicating the presence of subclinical infections. His initial magnetic resonance imaging was inconclusive, but the diagnosis was established following a lumbar puncture and subsequent cerebrospinal fluid analyses.

## Introduction

The herpes simplex virus (HSV) is the most often encountered viral cause of encephalitis [1]. It frequently shows clinically as behavioral abnormalities, cognitive impairment, and language impairment. While this is more usually caused by HSV-1, 2%-10% of cases of HSV encephalitis can be attributed to HSV-2. Primary HSV-2 infection occurs at the mucocutaneous surface and involves retrograde virus transfer to the peripheral sensory ganglia, with subsequent viral genome retention, and occasional reactivation through anterograde transmission to nerve terminals, it often manifests as painful ulcers, tender inguinal lymphadenopathy, dysuria, painful ejaculation in men and dyspareunia in females however, it may cause considerable neurological morbidity when it infects the central nervous system. We describe a case of an immunocompetent adult patient who was diabetic and had classic signs and symptoms of acute encephalopathy but no history of genital or oropharyngeal herpes. It's worth mentioning that HSV-2 has a seroprevalence of approximately 45 million cases in the United States, with more than 80% remaining asymptomatic, and an annual incidence of around 1 million new cases [2].

## Case presentation

**Figure 1:**
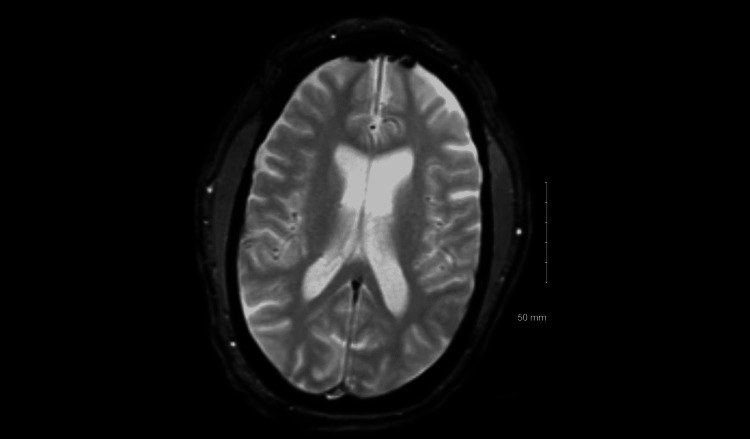
MRI of the head. Magnetic resonance imaging (MRI) did not show any evidence of edema, hemorrhage, infarct, hydrocephalus, or neoplasm.

We describe a 59-year-old male patient with a history of chronic inflammatory demyelinating polyneuropathy, congestive heart failure (CHF), diabetes mellitus, and nonalcoholic hepatic steatosis who arrived at the hospital with a two-day history of progressively worsening mentation, fever, and intermittent headaches. The patient had recently attended a wrestling match in a crowded venue and developed fever and headaches shortly thereafter. He was unable to provide a credible history during his initial presentation. The physical examination revealed a somnolent but restless patient who was arousable to verbal stimuli, could not provide detailed responses to questions, and had psychomotor slowdown; nonetheless, he did not seem to have dysarthria. His fluency, understanding, naming, and repetition were all intact. He exhibited hyporeflexia in the biceps, brachioradialis, patellar, and achilles tendon, a chronic finding associated with the patient's history of chronic demyelinating polyneuropathy. No assessment of sensation, coordination, or gait was possible. His initial vitals were remarkable for tachycardia, tachypnea, and fever. Laboratory findings showed mildly elevated lactic acid levels with elevated markers of inflammation and acute kidney injury (AKI) (Table 1). He was also taken for computed tomography (CT) scans of the head, chest, abdomen, and pelvis, which revealed no evidence of acute disease. Neurology was consulted, and the patient underwent an magnetic resonance imaging (MRI) of the brain that was unremarkable (Figure 1).

**Table 1:** Initial laboratory findings most suggestive of anion gap metabolic acidosis secondary to lactic acidosis. No significant abnormality could be appreciated on complete blood count, hepatic function panel. Slightly elevated erythrocyte sedimentation rate suggested underlying inflammatory process.

CBC WITH DIFFERENTIAL	Result	Reference range
White blood cell (WBC)	10.44	4.50 - 11.00 10*3/uL
Red blood cell (RBC)	5.04	4.50 - 6.30 10*6/uL
Hemoglobin	14.9	14.0 - 18.0 g/dL
Hematocrit	45	40 - 54 %
Mean corpuscular volume ( MCV)	90	82 - 101 fL
mean corpuscular hemoglobin (MCH)	29.6	27.0 - 34.0 pg
mean corpuscular hemoglobin concentration (MCHC)	33	32 - 36 g/dL
red blood cell distribution width (RDW)	14	11.5 - 14.5 %
Platelets	303	140 - 440 10*3/uL
mean platelet volume (MPV)	9.3	7.4 - 10.4 fL
Neutrophils %	76.5	38.0 - 70.0 %
Lymphocytes %	13.0	20.0 - 48.0 %
Monocytes %	7.8	4.0 - 12.0 %
Eosinophils %	0.7	0.0 - 6.0 %
Basophils %	1	0.0 - 2.0 %
Immature Granulocytes %	1	0.0 - 0.4 %
Absolute Neutrophils	8	1.7 - 8.0 10*3/uL
Lymphocytes Absolute	1.4	0.9 - 2.9 10*3/uL
Monocytes Absolute	0.8	0.3 - 0.9 10*3/uL
Eosinophils Absolute	0.1	0.1 - 0.5 10*3/uL
Basophils Absolute	0.1	0.0 - 0.3 10*3/uL
Immature Granulocyte Absolute	0.1	0.0 - 0.1
Hepatic function panel	Result	Reference range
Total Bilirubin	0.5	0.3 - 1.2 mg/dL
Bilirubin, Direct	0.2	0.0 - 0.5 mg/dL
Alkaline Phosphatase	70	40 - 150 U/L
aspartate aminotransferase (AST)	24	5 - 34 U/L
alanine transaminase (ALT/SGPT)	39	<=55 U/L
Total Protein	7.4	6.0 - 8.3 g/dL
Albumin	4.5	3.5 - 5.0 g/dL
Basic metabolic panel	Result	Reference range
Sodium	139	136 - 145 mmol/L
Potassium	4.3	3.5 - 5.1 mmol/L
Chloride	103	98 - 107 mmol/L
CO2	23	22 - 29 mEq/L
Anion Gap	13	5 - 14 mmol/L
Calcium	9.8	8.5 - 10.3 mg/dL
Blood urea nitrogen (BUN)	19	8 - 26 mg/dL
Creatinine	1.5	0.7 - 1.3 mg/dL
Estimated Glomerular Filtration Rate (eGFR)	48	>60 mL/min
Lactate	2.5	0.5 - 2.0 mmol/L
Procalcitonin	0.06	0.00 - 0.05 ng/mL
C-reactive protein (CRP)	0.5	0.0 - 0.8 mg/dL
erythrocyte sedimentation rate (ESR)	22	0 - 20 mm/hr

Due to the suspicion of meningitis, he underwent lumbar puncture for subsequent cerebrospinal fluid (CSF) studies, including culture and gram stain, Venereal Disease Research Laboratory test (VDRL), West Nile virus (WNV) IgG/IgM, Bartonella IgG/IgM, Parvovirus B19 IgG/IgM, and Lyme serology. Subsequently, the patient was started on an empiric meningitis regimen consisting of ceftriaxone, vancomycin, ampicillin, and acyclovir. The patient grew more agitated, necessitating transfer to the intensive care unit, where he was intubated and needed sedation with dexmedetomidine; he was also started on levetiracetam 500 mg twice daily for seizure prevention. Meanwhile, the panel for hepatitis and sexually transmitted diseases, including HIV, came out negative. However, HSV-2 was detected on the infectious disease panel on CSF (Table 2). The patient was diagnosed with HSV-2 encephalitis and remained on intravenous acyclovir, to which he responded and was subsequently extubated. His mental status steadily improved; he was eventually discharged on oral valacyclovir.

**Table 2:** Cerebrospinal fluid analysis findings showing clear CSF with lymphocytosis and positive HSV-2 PCR, and elevated Parvovirus B19 IgG antibodies suggesting prior infection. CSF: Cerebrospinal fluid, WBC: White blood cell, RBC: Red blood cell, VDRL: Venereal Disease Research Laboratory, LDH:  lactate dehydrogenase, HSV: Herpes simplex virus, PCR: polymerase chain reaction, B henselae: Bartonella henselae, B quintana: Bartonella quintana, Ab: antibody, IgG: Immunoglobulin G, IgM: Immunoglobulin M.

Cerebrospinal fluid analysis	Result	Reference range
Body Fluid Culture-Aerobic	No growth at 72 hours	
Body Fluid Culture-Anaerobic	No growth at 120 hours	
Gram Stain Result	Polymorphonuclear leukocytes, No Bacteria seen	
Color, CSF	Colorless	
Clarity, CSF	Clear	
WBC, CSF	382	<=5 /cumm
RBC, CSF	205	<=5 /cumm
Lymphocytes, CSF	98	%
Mono+Macro, CSF	2	<=37 %
VDRL CSF	Non-Reactive	Non-Reactive
LDH, Fluid	30	U/L
Cryptococcal Antigen CSF	Negative	Negative
Protein, CSF	172	15 - 45 mg/dL
Glucose, CSF	118	40 - 70 mg/dL
HSV 1, PCR	Negative	Negative
HSV 2, PCR	Positive	Negative
B henselae IgG	Negative	Neg:<1:320 titer
B henselae IgM	Negative	Neg:<1:100 titer
B quintana IgG	Negative	Neg:<1:320 titer
B Quintana IgM	Negative	Neg:<1:100 titer
Parvovirus B19 Ab, IgG, S	5.3	0.0 - 0.8 index
Parvovirus B19 Ab, IgM, S	0.2	0.0 - 0.8 index

## Discussion

As the name implies, encephalitis is a form of brain inflammation that is most often caused by a viral infection (particularly herpes simplex virus type 1 [HSV-1]) [1]. The majority of patients present with altered mental status, fever, and seizures, however uncommon movement disorders or localized neurological deficits may also be present and were absent in our patient. Although lumbar puncture and cerebrospinal fluid (CSF) evaluation are critical for diagnosis, imaging and electroencephalography (EEG) may also be useful. Many encephalitis patients have persistent physical or mental abnormalities that need long-term interdisciplinary therapy [1]. Although our patient was diabetic but immunocompetent, he had no history of genital or oropharyngeal herpes. As previously stated, the seropositivity for HSV-2 in the United States is estimated to be about 45 million cases with an annual incidence of one million cases. Encephalitis in or case is most likely the result of the virus becoming active after an asymptomatic infection in the past.

Despite the fact that no abnormalities were seen on magnetic resonance imaging of the brain in our case, for early detection of abnormalities, MRI is preferred over CT. The 100% sensitivity of MRI in identifying HSV encephalitis changes during the three- to 10-day interval after symptom onset supports its use as one of the criteria for excluding that diagnosis [3]. Electroencephalography is important in diagnosing and monitoring seizure activity, but is non-specific and may be abnormal in a variety of different types of encephalopathy [4]. Cerebrospinal fluid analysis is critical for establishing evidence of central nervous system (CNS) inflammation. Lumbar puncture (LP) is often performed overly late, especially to rule out elevated intracranial pressure. Not all patients need imaging prior to LP, and consensus recommendations propose a few specific criteria for imaging. If any of them are present, an immediate CT scan or, preferably, MRI should be done. It is necessary to do brain imaging for three reasons: to check for abnormalities indicative of encephalitis, to rule out other diagnoses, and to evaluate the patency of the basal cisterns and the lack of mass effect in order to continue with LP.

**Table 3:** Indications for neuroimaging before lumbar puncture (LP) in suspected encephalitis. The development of papilledema and focal neurological symptoms may suggest an increase in intracranial pressure or a mass effect that increases the risk of intracranial brain herniation following lumbar puncture.

Indications for neuroimaging before LP in suspected encephalitis [1].
1. Focal neurological signs.
2. Prescence of papilledema.
3. Glasgow coma scale (GCS) < 12

Lymphocytic pleocytosis (>5 white cells x 109/L) is a characteristic finding on CSF examination. However, neutrophils may be elevated early in the disease, or the white cell count may infrequently be normal [5]. Protein levels are normal to slightly elevated, while glucose levels are normal. In immunocompetent individuals, CSF polymerase chain reaction (PCR) for HSV has a sensitivity and specificity of more than 95% for HSV encephalitis [6]. When CSF is acquired relatively early in the illness course, PCR for HSV may be mistakenly negative in certain instances. Thus, if clinical suspicion of HSV persists, LP should be performed, which is often positive despite acyclovir therapy. Acyclovir treatment significantly improves the outcome of HSV encephalitis. In patients with suspected encephalitis, LP should be done quickly, followed by empirical therapy. If LP is delayed for six hours or longer, empirical aciclovir may be required [6]. Patients with HSV encephalitis will likely have PCR-positive CSF for a few days after starting acyclovir, hence LP should be done as soon as feasible. This will aid in diagnosis and hence guide tailor therapy.

Despite the fact that our patient had excellent neurological recovery with no persisting neurological abnormalities, many patients with encephalitis may have lasting physical and cognitive concerns and will need a comprehensive approach to their continued treatment.

## Conclusions

Even in the absence of genital complaints, HSV-2 should be recognized as a cause of encephalitis. MRI is the gold standard imaging modality for diagnosing HSV-2-associated encephalitis. It can help rule out other causes of encephalopathy, such as trauma or tumor, but can be non-contributory in some cases. The availability of CSF PCR assays has enhanced the capacity to establish quick diagnoses without delays in therapy. 
